# Relationship between pancreatic parenchyma loss and early postoperative hyperglycemia in patients with benign pancreatic diseases

**DOI:** 10.1007/s00261-021-03061-4

**Published:** 2021-04-04

**Authors:** Kan Wen, Chunyuan Cen, Leidi Wu, Mengting Huang, Hongli Yang, Xiaofei Yue, Yu Zhang, Guina Ma, Xin Li, Ping Han

**Affiliations:** 1 Department of Radiology, Union Hospital, Tongji Medical College, Huazhong University of Science and Technology, Wuhan, 430022 China; 2 Hubei Province Key Laboratory of Molecular Imaging, Wuhan, 430022 China

**Keywords:** Diabetes mellitus, Pancreatic surgery, Remnant pancreatic volume, Pancreatic endocrine insufficiency, CT imaging

## Abstract

**Objective:**

To evaluate the relationship between pancreatic parenchyma loss and early postoperative hyperglycemia in patients with benign pancreatic diseases.

**Methods:**

A total of 171 patients with benign pancreatic tumors or chronic pancreatitis, whose preoperative fasting blood glucose (FBG) was normal and who underwent partial pancreatectomy were reviewed. The pancreatic volume was measured by CT imaging before and after the operation. According to their different pancreatic resection volume (PRV), 171 patients were divided into five groups: < 30%, 30%–39%, 40%–49%, 50%–59%, and ≥ 60%. The correlation between the PRV and postoperative FBG was investigated. According to the postoperative FBG value, the patients were divided into a hyperglycemia group (HG) and nonhyperglycemia group (non-HG) to explore the best cutoff value of the PRV between the two groups.

**Results:**

There were significant differences in the postoperative FBG among the five groups (PRV < 30%, 30%–39%, 40%–49%, 50%–59%, and ≥ 60%). The PRV was positively correlated with postoperative FBG in the benign tumor group and chronic pancreatitis group (*R* = 0.727 and 0.651, respectively). ROC curve analysis showed that the best cutoff value of the PRV between the HG (*n* = 84) and non-HG (*n* = 87) was 39.95% with an AUC = 0.898; the sensitivity was 89.29%, and the specificity was 82.76%.

**Conclusion:**

There was a linear positive correlation between the postoperative FBG level and PRV. Patients with a PRV ≥ 40% are more likely to develop early postoperative hyperglycemia.

## Introduction

In recent years, the number of surgeries for benign pancreatic diseases has increased rapidly [1, 2]. On the one hand, the increased use of imaging modalities such as CT and MRI has led to an increase in the number of benign pancreatic tumors incidentally diagnosed [3, 4]. On the other hand, due to many risk factors, including alcohol consumption, biliary stones, and inherited characteristics, there is an increasing incidence of chronic pancreatitis [5, 6]. An increasing number of patients with chronic pancreatitis in whom medical treatments are ineffective or who present pseudocysts have undergone surgical treatment. The common surgical methods for benign pancreatic diseases are pancreaticoduodenectomy (PD), distal pancreatectomy (DP), duodenum-preserving pancreatic head resection (DPPHR), enucleation (EN), and central pancreatectomy (CP). These operations more or less resulted in the loss of pancreatic parenchyma.

The pancreas is an important endocrine organ in the human body. Loss of the pancreatic parenchyma directly leads to decreases in the numbers of multiple endocrine cells (alpha cells, beta cells, gamma cells, and PP cells) and affects the secretion of hormones such as insulin, glucagon, somatostatin, and pancreatic polypeptides, thus affecting the regulation of blood glucose by the pancreas [7]. Endocrine cells are distributed in the pancreas in the form of islets, which account for approximately 4.49% of the total pancreatic volume (0.94% in head, 0.97% in neck, 1.41% in body, 1.41% in tail), and beta-cell mass accounts for 60% of the islets [8]. The mechanism of endocrine insufficiency after pancreatic surgery has not been thoroughly studied, especially with regard to the relationship between quantitative pancreatic parenchyma loss and postoperative fasting blood glucose (FBG). Several studies have discussed the compensatory capacity of residual pancreatic parenchyma. Maignan A et al., Kwon JH et al., and Singh AN et al. believe that pancreatic parenchyma loss is a risk factor for postoperative hyperglycemia [9–11].Conversely, King J et al. stated that retaining 20% to 25% of the pancreatic parenchyma can maintain pancreatic function [12], and surgical resection of portions of the pancreatic parenchyma will not affect endocrine function.

The postoperative survival time of patients with pancreatic malignant tumors is limited, and for patients with malignant tumors, the first consideration is tumor section followed by preservation of pancreas function. For patients with curable and long-lived benign pancreatic diseases, it is necessary to explore the effect of pancreatic parenchyma loss on postoperative blood glucose. Postoperative hyperglycemia may lead to long-term complications and a decline in the quality of life in these patients. In this study, our aim was to measure pancreatic volume by CT imaging and to explore the relationship between pancreatic parenchyma loss and postoperative blood glucose to provide some reference for the maintenance of pancreatic function after partial pancreatectomy.

## Methods

### Patients

**Fig. 1:**
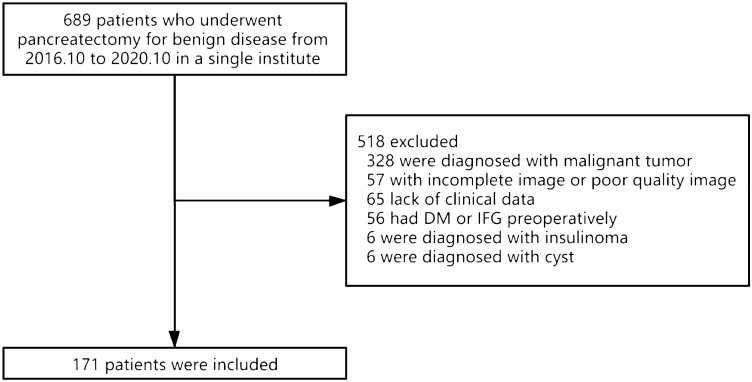
Flow diagram of patient enrollment

In this study, retrospective data from patients who underwent partial pancreatectomy in our hospital from October 2016 to October 2020 were collected. The inclusion criteria were as follows: (1) resection of the pancreatic parenchyma; (2) plain and contrast-enhanced CT images before and two week after the operation; (3) available pathological results provided after the operation; (4) no use of hypoglycemic drugs or insulin; and (5) no use of hormones, antipsychotics, or antitumor drugs. The exclusion criteria were as follows: (1) poor image quality that could not be used for analysis; (2) pathological results indicative of malignant tumors; (3) functional neuroendocrine tumors; and (4) diagnosed diabetes mellitus or impaired fasting blood glucose before surgery (Fig. 1).

A total of 171 patients met the above criteria: 37 with solid pseudopapillary tumors, 35 with intraductal papillary mucinous tumors, 14 with mucinous cystadenomas, 7 with serous cystadenomas (suspected to be malignant or have related symptoms), 22 with nonfunctional neuroendocrine tumors of the pancreas, 3 with schwannomas, and 53 with chronic pancreatitis (either unresponsive to medical treatment or presenting pseudocysts).

This study was approved by the ethics committee of our institution. CT examinations were performed for preoperative diagnosis and postoperative evaluation of recovery. Blood glucose measurement is a parameter included in clinical biochemistry blood tests. This study only collected these results, did not interfere with the patient’s treatment plan, and did not divulge the patients’ personal information.

### Evaluation of endocrine function before and after the operation

The blood glucose levels of the patients during the hospitalization period were statistically analyzed. The average FBG level 7 days before the operation was taken as the preoperative FBG value (results of 2 to 5 tests over 7 days), and the average FBG value 14 days after the operation was defined as the postoperative FBG value (results of 5 to 10 tests in 14 days: 1, 2 and 3 days after operation and every 2–3 days after that).

According to the postoperative FBG, the patients were divided into three groups. FBG ≥ 126 mg/dL (7.0 mmol/L) was classified as the hyperglycemic group (HG), FBG 110 mg/dL–126 mg/dL (6.1 mmol/L–7.0 mmol/L) was classified as the impaired fasting blood glucose group (IFG), and FBG < 110 mg/dL (6.1 mmol/L) was classified as the normal glycemia group (NG).

### Calculation of the volume loss of the pancreas

CT was performed with a Siemens SOMATOM Definition AS+ 128 CT scanner. The voltage of the tube was 120 kV, the tube current adopted intelligent current regulation technology, the pitch was 1.0, the width of the collimator was 0.6 mm×128, the layer thickness was 2.0 mm, the layer spacing was 2.0 mm, and the visual field was 390×390 mm. The enhanced scan was performed with a nonionic iodine contrast agent (350 mg I/ml), which was injected into the median cubital vein with a high-pressure syringe at a flow rate of 2.5 ml/s and a total volume of 1.0 ml/kg. The arterial phase, portal phase, and delayed phase were scanned 25–30 s, 55–65 s, and 150–180 s after injection, respectively.

Measurement of the pancreatic volume was performed on a Siemens syngo.via postprocessing workstation. Portal venous phase images from the enhanced CT scans were selected for measurement. At this time, the enhancement of the pancreas was obvious, the boundary was clear, and the contrast between normal pancreatic tissue and the tumor, surrounding fat, and peripheral blood vessels was clear, which could effectively avoid interference.

**Fig. 2:**
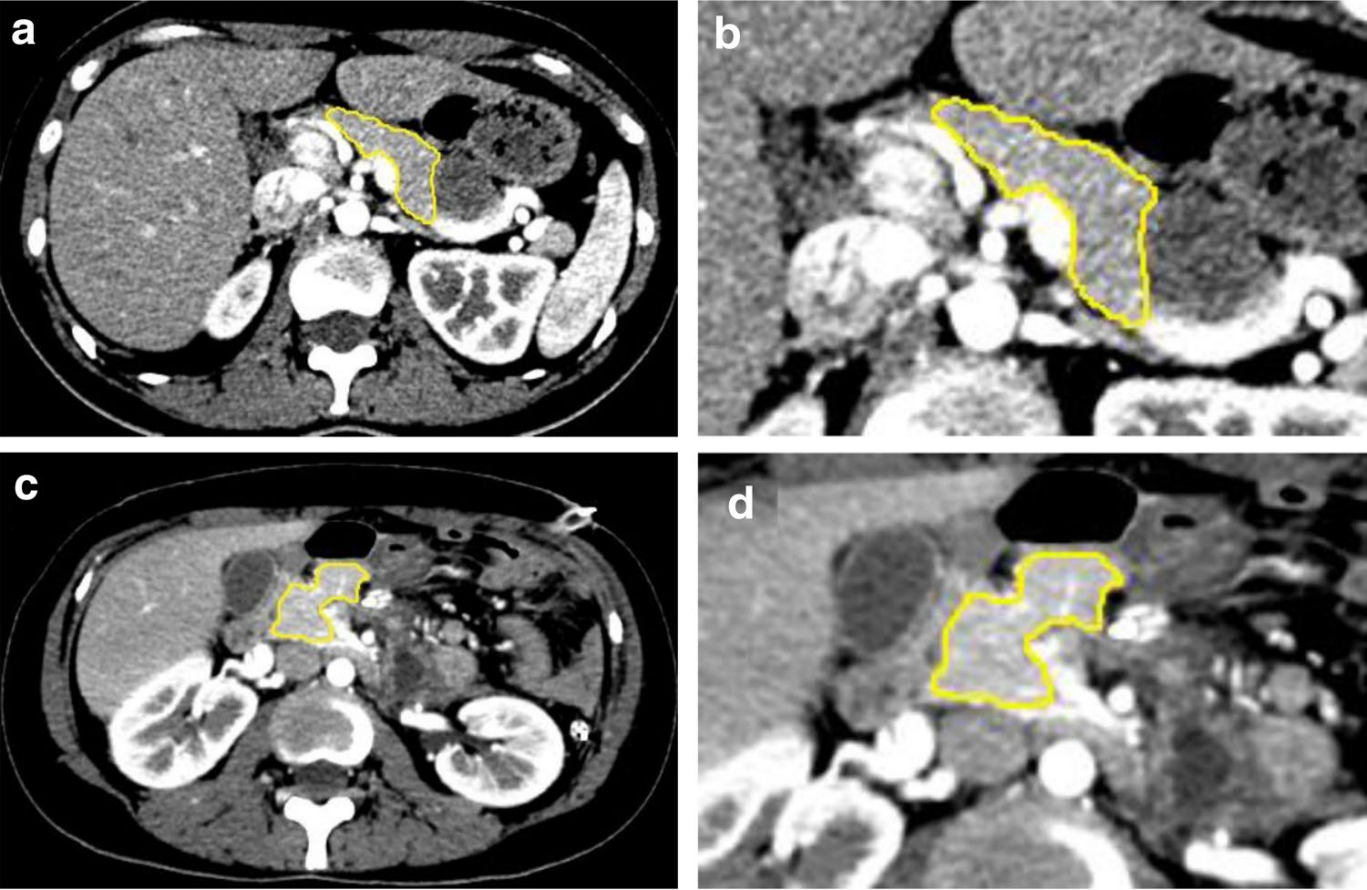
**a**–**d** A schematic diagram of the volume of the pancreas. Female, 30 y, with a solid pseudopapillary tumor pathologically, underwent distal pancreatectomy and splenectomy. **a** Preoperative CT image. **b** A locally magnified picture of a region from (**a**). **c** Postoperative CT image. **d** A locally magnified picture of a region from (**c**). The volume of the pancreas was 52.45 cm^3^ before the operation and 31.88 cm^3^ after the operation

The outline of the pancreas of the patient was drawn layer by layer (layer thickness = 2 mm) to avoid peripheral blood vessels, surrounding organs, tumors (including the cystic part and the solid part), stones (larger than 5 mm), calcification (larger than 5 mm), inflammatory exudation, postoperative necrosis, etc., and only the enhanced pancreatic parenchyma was outlined (Fig. 2). Through the three-dimensional volume of interest (VOI) measurement tool, the preoperative and postoperative pancreatic volume (accurate to 0.01 cm^3^) was automatically calculated according to the delineated area and layer thickness. The volume of the pancreas was measured by two radiologists with 3-5 years of working experience who were blinded to the patients’ clinical information, and the average value of the measured results was taken as the final result. The uniformity between the two radiologists was tested using the intraclass correlation coefficient (ICC).

The following equations were used to calculate the relevant parameters: PRV (cm^3^) = preoperative pancreatic volume (cm^3^) - postoperative pancreatic volume (cm^3^) and PRV ratio = (1-postoperative pancreatic volume/preoperative pancreatic volume) × 100%. According to the PRV, 171 patients were divided into 5 groups based on resection volume: < 30%, 30%–39%, 40%–49%, 50%–59%, and ≥ 60%.

### Statistical analysis

A paired-sample t test was used to compare FBG before and after the operation. The preoperative and postoperative blood glucose levels of patients with benign pancreatic tumors and chronic pancreatitis were compared by independent-sample t tests. The postoperative blood glucose levels of patients with different PRVs were tested by the F test. The correlation between the PRV and blood glucose level was analyzed. Receiver operating characteristic (ROC) curves were used to analyze the best cutoff value, sensitivity, and specificity of the PRV among the different postoperative blood glucose groups. The chi-square test was performed on a variety of clinical factors in the HG, IFG, and NG. *P* < 0.05 was considered to be statistically significant.

## Results

### Blood glucose level of patients before and after operation

Among the 171 patients, 76 were male and 95 were female, with an average age of 49.1 ± 13.3 years. A total of 71 patients underwent DP, 61 patients underwent PD, 25 patients underwent DPPHR, 11 patients underwent EN, and 3 patients underwent CP.

There was no significant difference in preoperative FBG between the benign pancreatic tumor group and the chronic pancreatitis group (*P* = 0.162). However, there was a significant difference in postoperative FBG between the benign pancreatic tumor group and the chronic pancreatitis group (*P* = 0.010). The postoperative FBG in the chronic pancreatitis group was higher than that in the benign pancreatic tumor group. There was a significant difference in FBG before and after the operation (*P* < 0.001) and the blood glucose level after surgery was higher than that before surgery (Table 1).

**Table 1:** Comparison of FBG before and after operation in patients with benign pancreatic tumor and chronic pancreatitis (mg/dL)

	Preoperative FBG	Postoperative FBG	P value
benign pancreatic tumor (*n* = 118)	90.54 ± 11.70	128.16 ± 23.76	< 0.001
chronic pancreatitis (*n* = 53)	93.24 ± 12.96	142.74 ± 36.18	< 0.001
P value	0.162	0.010	

### Postoperative blood glucose levels with different PRVs

**Fig. 3:**
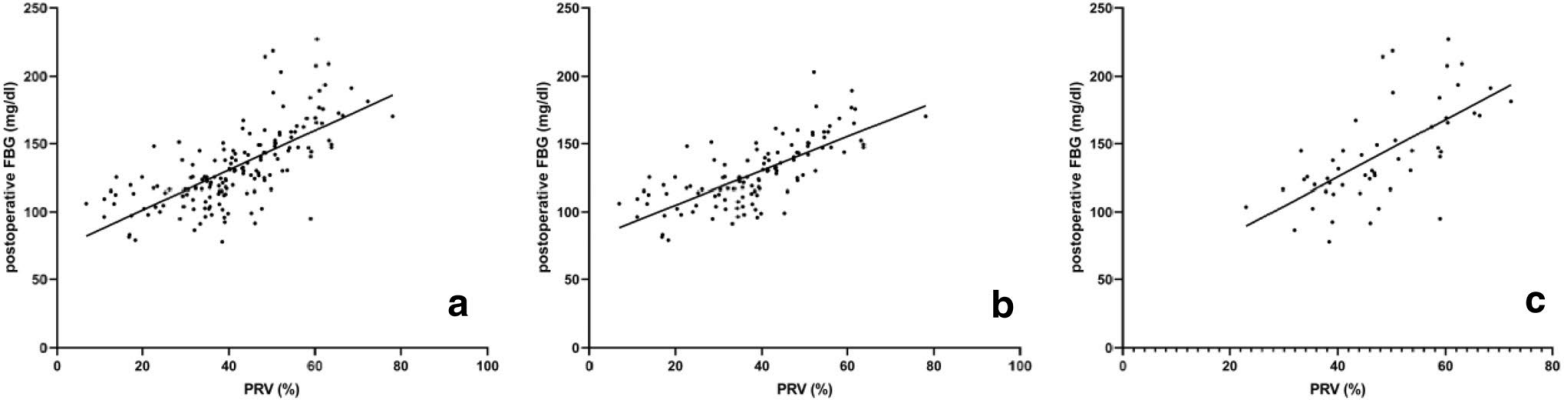
**a** Correlation analysis between PRV and postoperative FBG in 171 patients with benign pancreatic diseases, *R* = 0.691, *P* < 0.001. **b** Correlation analysis between PRV and postoperative FBG in 118 patients with benign pancreatic tumor, *R* = 0.727, *P* < 0.001. **c** Correlation analysis between PRV and postoperative FBG in 53 patients with chronic pancreatitis, *R* = 0.651, *P* < 0.001

The postoperative blood glucose levels of the five groups of patients with a PRV < 30%, 30%–39%, 40%–49%, 50%–59%, and ≥ 60% were compared (Table 2), and there was a significant difference among the five groups. The correlation analysis between the PRV and postoperative blood glucose in the benign tumor and chronic pancreatitis groups showed that there was a positive linear correlation (*R* = 0.727 and 0.651, respectively; *P* < 0.001 for both) (Fig. 3).

**Table 2:** Postoperative blood glucose levels with different PRVs (mg/dL)

PRV	*N*	Benign pancreatic tumor (*n* = 118)	N	Chronic pancreatitis (*n* = 53)
< 30% (group A)	28	110.34 ± 17.46	2	110.16 ± 9.00
30%–39% (group B)	38	115.74 ± 13.14	13	114.12 ± 19.44
40%–49% (group C)	27	135.54 ± 13.32	16	133.02 ± 28.26
50%–59% (group D)	17	156.42 ± 16.38	12	153.72 ± 31.50
≥ 60% (group E)	8	165.78 ± 15.12	10	188.82 ± 20.52
*P* value		< 0.001		< 0.001

Pairwise comparison: (1) Postoperative FBG in benign tumor group: there was significant difference between group A and groups C, D, and E, between group B and groups C, D, and E, between group C and groups A, B, D, and E, between group D and groups A, B, and C, and between group E and groups A, B, and C (*P* < 0.05). (2) Postoperative FBG in chronic pancreatitis group: there were significant differences between group A and groups D and E, between group B and groups D and E, between group C and groups D and E, between group D and groups A, B, C, and E, and between group E and groups A, B, C, and D (*P* < 0.05)

### Comparison among the HG, IFG and NG

According to postoperative FBG, 171 patients were divided into three groups: HG (*n* = 84), IFG (*n* = 52) and NG (*n* = 35). As shown in Table 3, there was no significant difference in sex, age, hypertension, hyperlipidemia, BMI, or operation type among the three groups.

**Table 3:** Comparison of clinical factors among HG group, IFG group, and NG group

	All (*n* = 171)	HG group (*n* = 84)	IFG group (*n* = 52)	NG group (*n* = 35)	*P* value
Sex (female)	95/171	52/84	27/52	16/35	0.221
Age	49.1 ± 13.3	50.0 ± 11.7	49.0 ± 13.8	47.3 ± 16.2	0.605
Hypertension	38/171	20/84	12/52	6/35	0.716
Hyperlipidemia	41/171	20/84	13/52	8/35	0.973
BMI ≥ 25.0 kg/m^2^	29/171	16/84	8/52	5/35	0.767
Operation type					0.563
PD	61/171	29/84	18/52	14/35	–
DP	71/171	35/84	23/52	13/35	–
DPPHR	25/171	15/84	4/52	6/35	–
EN	11/171	4/84	5/52	2/35	–
CP	3/171	1/84	2/52	0/35	–

*PD* pancreaticoduodenectomy, *DP* distal pancreatectomy, *DPPHR* duodenum-preserving pancreatic head resection, *EN* enucleation, *CP* central pancreatectomy

### ROC curve analysis

**Fig. 4:**
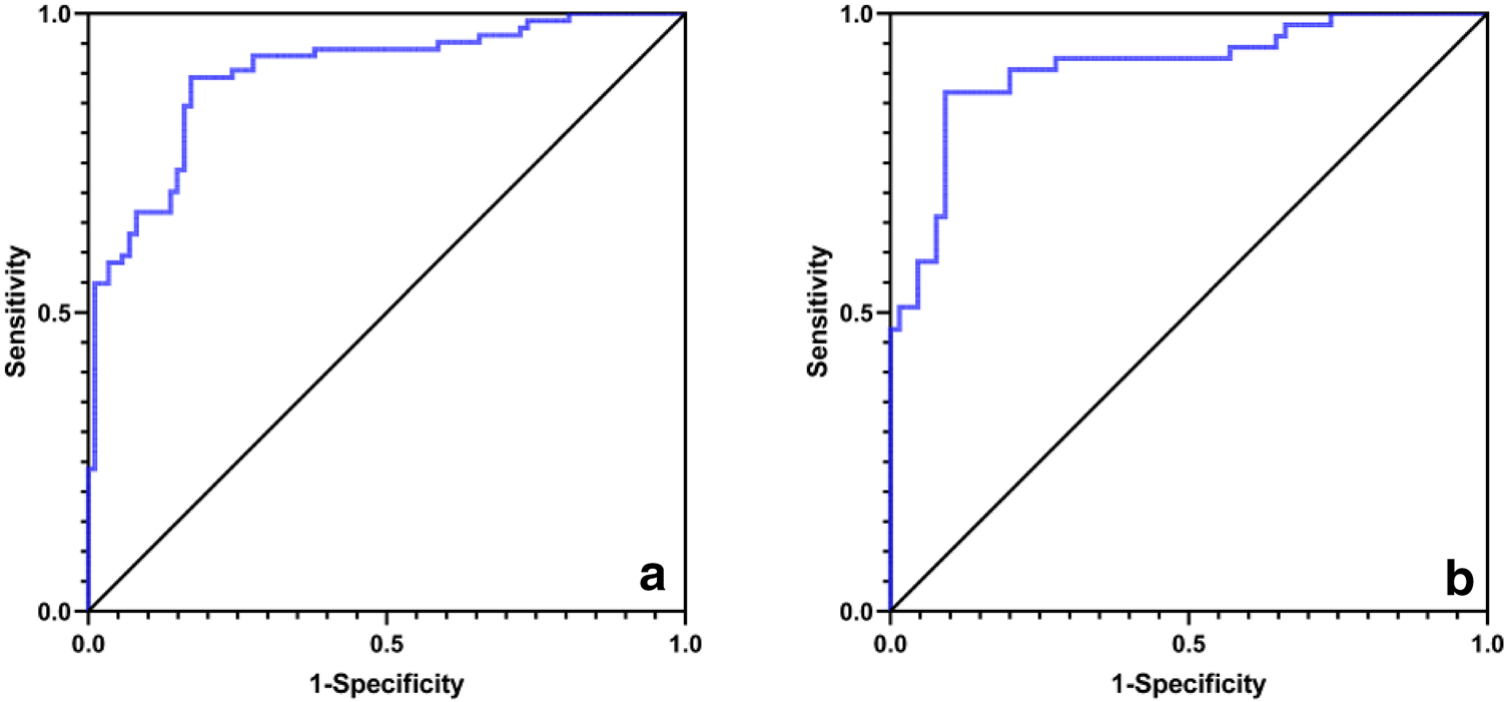
**a** ROC curve of the HG group and non-HG group in 171 patients with benign pancreatic diseases, AUC = 0.898, *P* < 0.001; **b** ROC curve of the HG group and non-HG group in 118 patients with benign pancreatic tumor, AUC = 0.909, *P* < 0.001

In 171 patients with benign pancreatic lesions, the average PRV of the HG, IFG, and NG was 50.55% ± 10.26%, 34.54% ± 9.35%, and 30.07% ± 11.79%, respectively. The difference among the three groups was statistically significant (*F* = 65.495, *P* < 0.001). The IFG and NG were combined to form the nonhyperglycemia group (non-HG). ROC curve analysis showed that the best cutoff value of the PRV between the HG and non-HG was 39.95% with an area under the curve (AUC) of 0.898 (*P* < 0.001); the sensitivity was 89.29%, and the specificity was 82.76% (Fig. 4A).

In 118 patients with benign pancreatic lesions, the average PRV of the HG was 48.56% ± 10.26%, that of the IFG was 32.53% ± 9.66%, and that of the NG was 27.12% ± 10.49%. The difference among the three groups was statistically significant (*F* = 49.868, *P* < 0.001). ROC curve analysis showed that the best cutoff value of the PRV between the HG and non-HG was 39.99% with an AUC of 0.909 (*P* < 0.001); the sensitivity was 86.79%, and the specificity was 90.77% (Fig. 4B).

## Discussion

### Glycometabolic state before and after resection

From Table 1, in patients with normal preoperative glucose metabolism, blood glucose increased to varying degrees after partial pancreatectomy, and there was a statistically significant difference before and after surgery (*P* < 0.001). When comparing the benign tumor and chronic pancreatitis groups, we found that there was a statistically significant difference in postoperative FBG with the chronic pancreatitis group presenting a higher postoperative FBG. One possible reason is that benign tumors of pancreas may have no adverse effect on the background pancreatic parenchyma, and the compensatory ability of the residual pancreatic parenchyma is strong after partial pancreatectomy, while chronic pancreatitis, as an irreversible chronic inflammatory disease of the pancreas, may also affect the function of the residual pancreas, thus affecting the regulation of blood glucose after surgery [13, 14].

### The PRV and the postoperative glycometabolic state

Due to the high variability in the locations of pancreatic tumors, the selected procedure and scope of resection also differ. The chosen operation will reduce the pancreas parenchyma while removing the lesion. Pancreatic glands are nonrenewable and pancreatic parenchyma loss directly leads to the loss of many kinds of endocrine cells and impacts the secretion of hormones, thus affecting pancreatic-mediated regulation of blood glucose [7]. Several studies have discussed the compensatory capacity of residual pancreatic parenchyma after partial pancreatectomy. King J et al. believe that 20% to 25% of the pancreatic parenchyma can maintain pancreatic function [12], and the pancreatic parenchyma loss due to surgical resection will not affect the endocrine function of the pancreas. Leal JN et al. compared 103 patients who underwent partial pancreatectomy with 31 patients who did not undergo surgery and concluded that the operation did not affect the probability of new-onset diabetes [15]. Shirakawa S et al. concluded that a PRV > 44% was an independent risk factor for postoperative new-onset diabetes. They divided 38 patients into three groups based on PRV(< 25%, 25%–50%, and > 50%) and found that the rates of new-onset diabetes were 34.3%, 53.3%, and 73.3%, respectively [16]. Kwon JH et al. reported that there was a high incidence of postoperative diabetes in patients whose volume ratio of pancreatectomy was greater than 35.6% [10]. Izumo W et al. showed that in the preoperative low-risk group, the cutoff value of the PRV was 42.1% [17].

In the study, we found that patients with a PRV ≥ 40% were more likely to develop early postoperative hyperglycemia (AUC = 0.898, *P* < 0.001), which was consistent with the cutoff value of new-onset postoperative diabetes in the abovementioned study. At the same time, we also found that there were significant differences in postoperative FBG levels in the different PRV groups (< 30%, 30%–39%, 40%–49%, 50%–59%, and ≥ 60%), and the correlation analysis between the PRV and postoperative FBG indicated that there was a positive linear correlation.

These data show that the PRV is closely related to postoperative blood glucose in patients with benign pancreatic lesions. In recent years, several studies have compared the effectiveness of segment pancreatectomy with traditional PD or DP in the treatment of benign or low-grade malignant tumors. Segment pancreatectomy can maximize the preservation of normal pancreatic parenchyma and minimize the effect of pancreatic parenchyma loss on pancreatic endocrine function [18].

In the study by Wu et al., it was found that the incidence of diabetes was 16% after PD (95% CI: 14%–17%), 21% after DP (95% CI: 16%–25%), and 6% after CP (95% CI: 3%–9%) [19]. Compared with other types of pancreatectomy, CP had a much lower incidence of DM. Beger HG et al. found that EN, CP, and DPPHR can better preserve pancreatic function in benign tumor surgery [20] whereas Zhou et al. reported that the incidence of pancreatic endocrine dysfunction after EN was lower than that after PD and DP [21]. The PRV has a direct effect on pancreatic endocrine function, and measuring pancreatic volume can predict the change trend of postoperative FBG.

In the pancreas of healthy adults, islets are mainly distributed in the body and tail [8]. In fact, DP may result in an approximately 70% loss of β-cell mass, while PD results in a nearly 30% loss. This is consistent with the results of Wu L et al., who found that the incidence of diabetes after DP was higher than that after PD [19]. Postoperative hyperglycemia is a multifactorial process. Wu L et al. considered that the risk of developing T3cDM is associated with the type of surgery, the PRV and a higher preoperative blood glucose [19]. Ko SW et al. stated that PD was a significant factor for endocrine insufficiency (HR = 3.87, 95% CI: 1.12–14.66, *P* = 0.037) [22]. In our study, the effect of surgery type on early postoperative hyperglycemia was not statistically significant in 117 patients with benign pancreatic diseases. There are many reasons contributing to this, the primary of which may be the insufficient number of cases undergoing procedures with small resection volumes, such as DPPHR, EN, and CP. This may be because the total number of cases is not large enough to show all the influencing factors. In addition, long-term follow-up of this cohort may reveal more information. In this study, patients with DM and IFG were excluded to control for preoperative confounding factors, which may be the reason why there was no difference in preoperative blood glucose among the groups.

This study is a single-center retrospective study in which the long-term blood glucose index values of the patients were not available, so it was unable to analyze longitudinal changes in glucose metabolism after the operation, which limits our interpretation of the results. At the same time, because the number of patients with chronic pancreatitis undergoing surgery is limited, they were not divided into separate groups in the analysis of postoperative blood glucose. Therefore, extending the postoperative follow-up time and further expanding the number of cases will be goals for our next study.

### Conclusion

In summary, there was a positive linear correlation between the postoperative early blood glucose level and the PRV in patients with benign pancreatic lesions. Furthermore, patients with a PRV ≥ 40% were more likely to develop early postoperative hyperglycemia.
